# Recurrent Ileocolic Intussusception With the Appendix as the Pathologic Lead Point in Children: A Report of Two Cases and Review of Literature

**DOI:** 10.7759/cureus.61120

**Published:** 2024-05-26

**Authors:** Nikita R Peramsetty, Tiffany Fung, Andi Zhang, Christian Saliba, Christopher Blewett, Shin Miyata, Richard Herman

**Affiliations:** 1 Pediatric Surgery, Saint Louis University School of Medicine, St. Louis, USA; 2 Pediatric Surgery, SSM Health Cardinal Glennon Children's Hospital, Saint Louis, USA

**Keywords:** dilated appendix, nonoperative reduction, surgical reduction, appendiceal intussusception, recurrent ileocolic intussusception

## Abstract

Ileocolic intussusception is a consideration in young pediatric patients with acute abdominal pain. Meckel’s diverticulum is the most common pathologic lead point for intussusception in children and the appendix acting as the lead point is rare. In addition, management guidelines for recurrent ileocolic intussusception (RICI) are lacking. We present two cases of RICI in which the pathological lead point was the appendix.

The first patient, a two-year-old with no medical history, had intermittent abdominal pain and non-bloody vomiting for a month. Ultrasound revealed ileocolic intussusception, successfully managed with pneumatic reduction. However, symptoms recurred and a repeat ultrasound showed partial intussusception of the appendix into the cecum. Laparoscopic reduction and appendectomy were then performed. Symptomatic intussusception recurred, and a second laparoscopic reduction with stump appendectomy resolved all symptoms. The second patient, a three-year-old with no medical history, had colicky abdominal pain for 24 hours. Ultrasound revealed ileocolic intussusception that was pneumatically reduced. As pain recurred, laparoscopic reduction and appendectomy were performed, revealing ileocolic intussusception with a dilated appendix as the pathologic lead point.

Recurrent ileocolic intussusception (RICI) with the appendix as the lead point is common, but RICI with the appendix as the lead point is rare. These cases demonstrate the role of the appendix as a pathologic lead point, and a review of the literature supports the need for surgical reduction. While enema reduction is the first line for recurrent intussusception, surgical reduction is preferred when a pathological lead point is suspected.

## Introduction

Ileocolic intussusception (ICI) is considered the second most common cause of acute bowel obstruction in children, the first being pyloric stenosis [1]. The incidence of pediatric ICI in the United States is approximately 56 cases per 100,000 children per year [2]. While ICI is common, the appendix as the lead point for intussusception is rare. Intussusception occurs when a more proximal portion of the bowel invaginates into a more distal part of the bowel. This can lead to venous compression and bowel wall edema, eventually leading to bowel ischemia, necrosis, perforation, and potentially death if not promptly addressed [3]. The majority of ICI cases are idiopathic, as seen in patients with no other pathological factors other than lymphoid hyperplasia at the terminal ileum. On the other hand, ICI can also be due to identifiable secondary causes, also known as lead points, the most common of which are Meckel’s diverticulum, intestinal polyps, and intestinal tumors (benign and malignant) [4]. While Meckel’s diverticulum is the most common lead point for intussusception in children, the appendix as the lead point is much rarer and there is a paucity of cases described in literature [5,6].

First-line treatments of ICI are enema reductions with pneumatic (air) or hydrostatic (saline, barium) options, due to their high success rate (70-80% on average) and low rates of complications [3,7,8]. Surgical treatment is typically reserved for pathological lead point (PLP), failed non-operative intervention, and signs of bowel perforation or hemodynamic instability. However, a significant portion of reductions do recur, reported to be around 20%, and is the most common complication after successful nonoperative reduction [9]. Previous studies on recurrent ileocolic intussusception (RICI) have mainly focused on the clinical and ultrasound aspects of diagnosis and calculation of risk factors that predispose one to RICI [10-12], while the guidelines for RICI management remain unclear. Some authors suggest surgical intervention after more than one episode of ICI, while others recommend a second round of nonoperative reduction with air or barium enema [13,14]. We present two cases of RICI with the appendix as the pathological lead point and a review of the literature.

## Case presentation

Case 1

**Figure 1:**
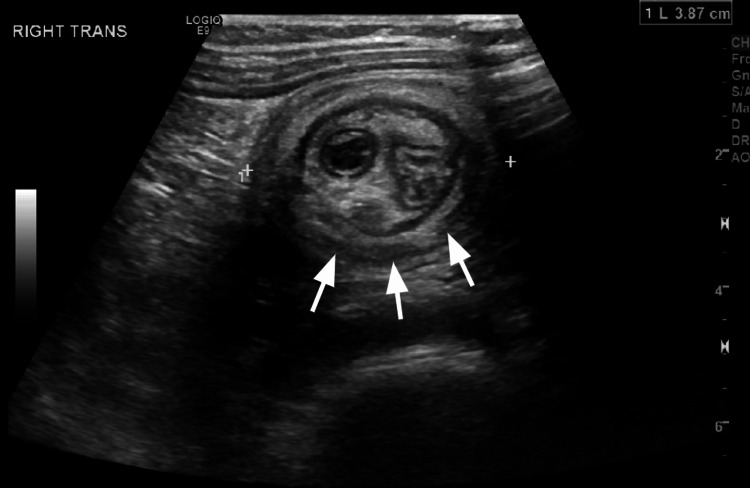
Abdominal ultrasound image The image shows ileocolic intussusception with a dilated appendix measuring 8 mm in diameter.

A two-year-old male with no previous past medical history presented with intermittent abdominal pain, non-bloody, nonbilious emesis, after meals for the past month. An initial abdominal ultrasound (US) revealed ileocolic intussusception and a dilated appendix (Figure 1). After successful treatment with an air enema, he was discharged the following morning.

**Figure 2:**
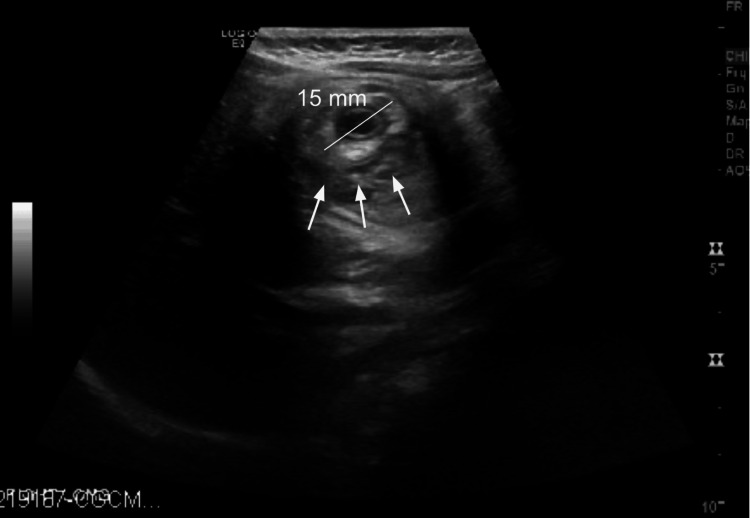
Abdominal ultrasound image on the second presentation The image shows recurrent ileocolic intussusception with a dilated appendix measuring 15 mm in diameter.

He represented that evening with abdominal pain, non-bloody, nonbilious emesis where an ultrasound revealed a recurrent ileocolic intussusception, again with a dilated appendix now measuring 15 mm in diameter (Figure 2). A repeat air enema was unable to confirm successful reduction, and subsequent ultrasound showed persistence of the intussusception. The repeat US also showed bowel wall thickening and increased echogenicity of adjacent mesentery and reactive, rounded mesenteric lymph nodes. The decision was then made to proceed with surgical intervention.

Intraoperatively, the intussusception was successfully reduced laparoscopically. The bowel was healthy and viable, however the appendix was dilated, edematous, and demonstrated a tendency to invaginate back into the cecum. An appendectomy was performed because of those findings. Pathology of the appendix showed a 0.3 cm diameter lumen with no distinct lesion. On microscopic evaluation, the ganglionated appendix with intact lymphoid tissue in its wall had no evidence of mucosal ulceration, luminal pus, transmural neutrophilic inflammation, or serositis. Postoperatively, he initially struggled with PO intake but was discharged on postoperative day (POD) two in stable condition. 

**Figure 3:**
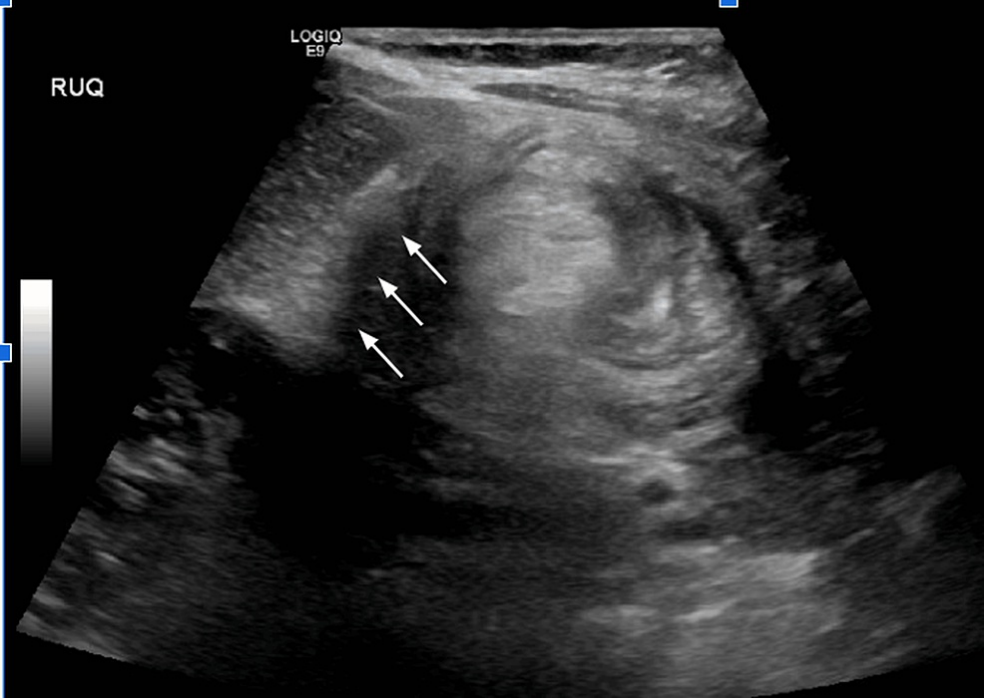
Abdominal ultrasound image four days after the first surgery The image shows recurrent ileocolic intussusception to the level of the hepatic flexure (white arrow).

**Figure 4:**
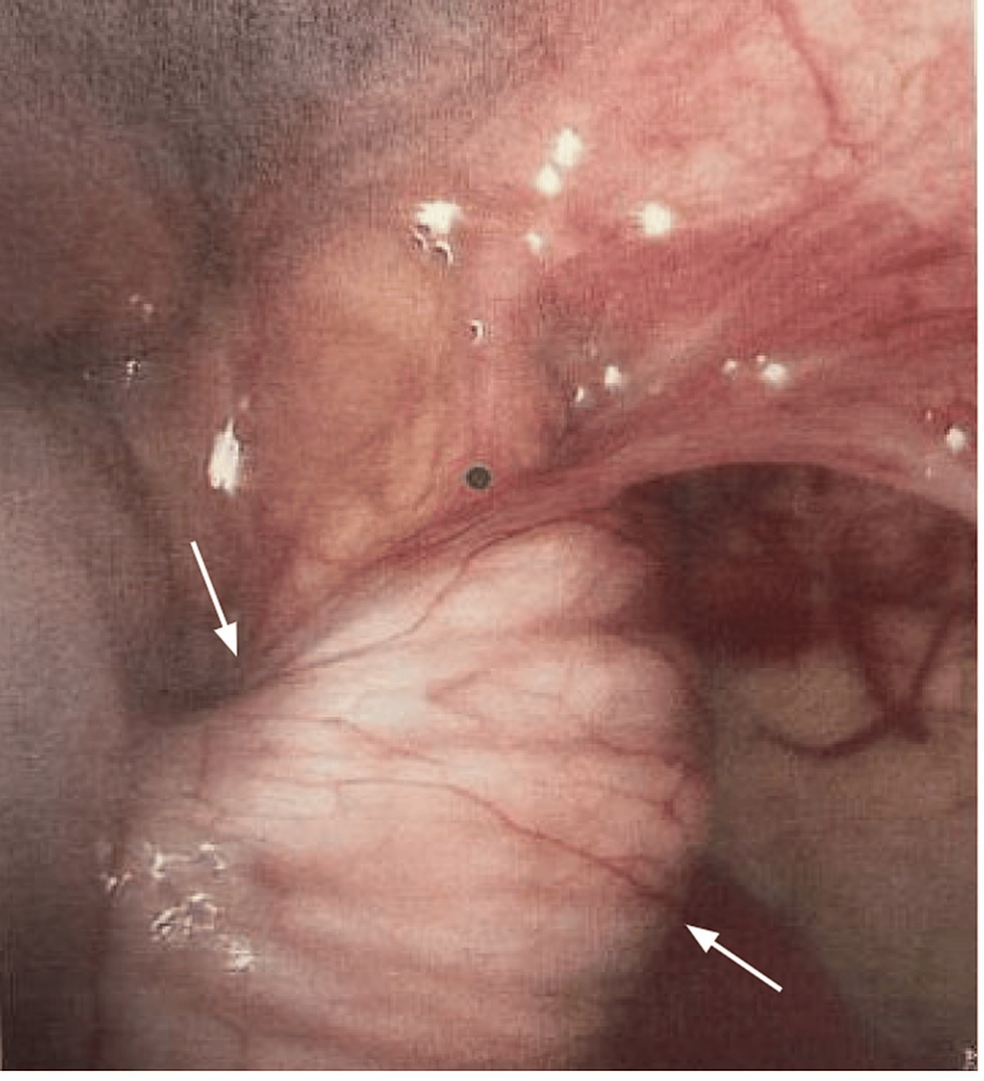
Intraoperative image one during the second surgery The arrows show the ileocecal intussusception.

**Figure 5:**
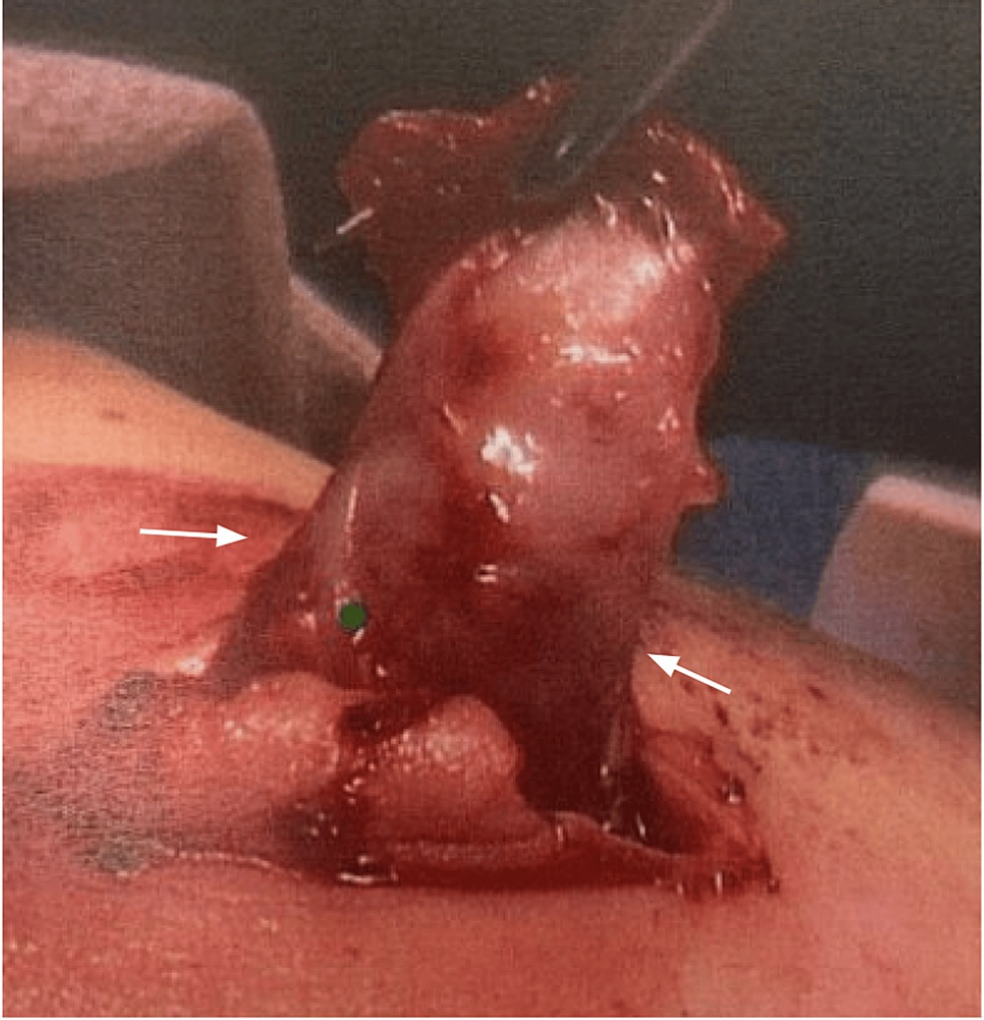
Intraoperative image two during the second surgery The image is showing a 2 cm appendiceal stump acting as a lead point.

However, he returned to the emergency department (ED) on POD four with episodic abdominal pain like the initial ED visit, again associated with non-bloody, nonbilious emesis. Ultrasound showed recurred ileocolic intussusception to the hepatic flexure (Figure 3). The decision was made to proceed directly to the operation room (OR) for diagnostic laparoscopy. Intraoperatively, the bowel was again easily reduced and was viable and healthy (Figure 4). However, a large appendiceal stump, approximately 2 cm, was encountered (Figure 5). A stump appendectomy was performed. The appendiceal stump was suspected to have acted as a lead point for the recurrent intussusception. Pathology on the appendiceal stump revealed chronic inflammation and follicular hyperplasia. Postoperatively, he did well and was discharged on POD two.

Case 2 

**Figure 6:**
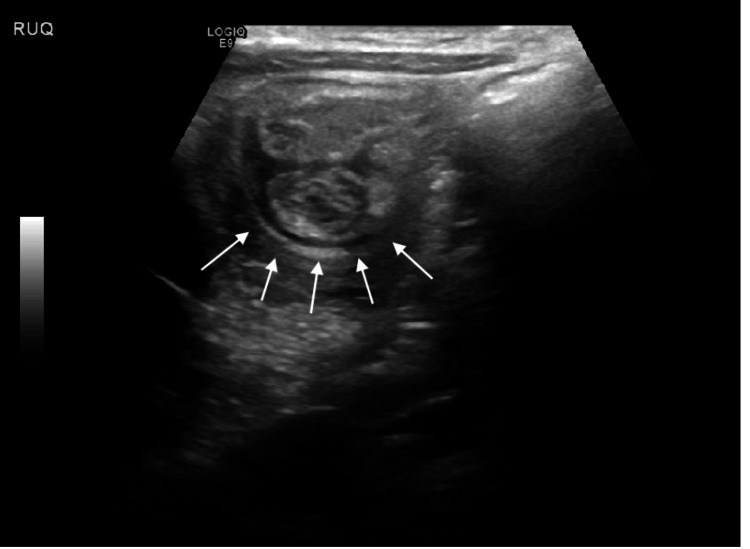
Abdominal ultrasound image at the time of presentation The image shows Ileocolic intussusception at least to the level of the hepatic flexure (white arrows).

**Figure 7:**
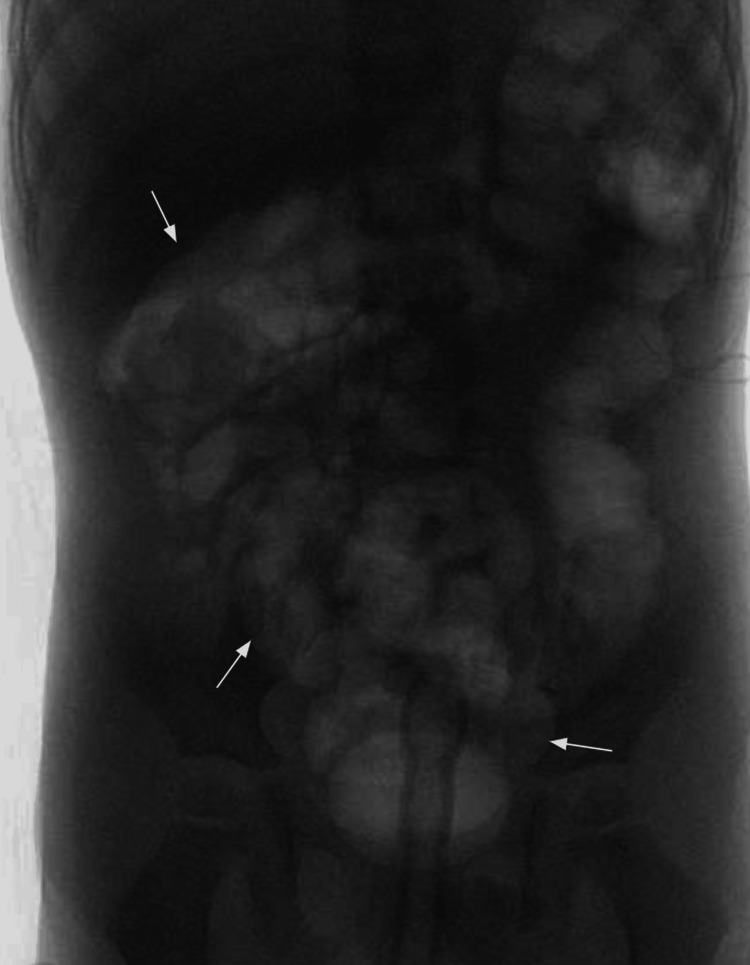
Lower gastrointestinal (GI) fluoroscopy image after air enema application The image shows successful ileocolic intussusception reduction via air enema.

**Figure 8:**
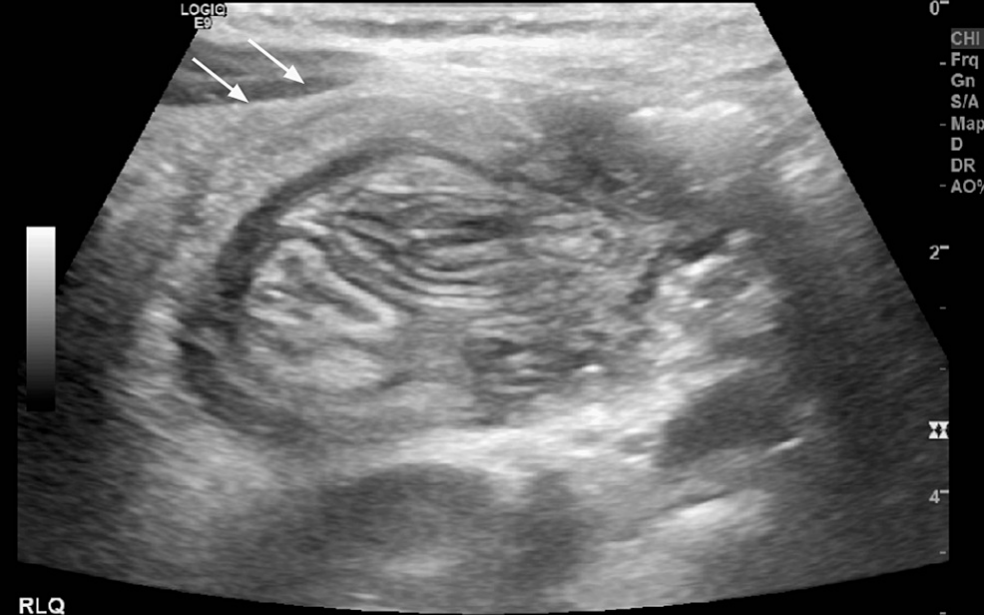
Abdominal ultrasound image one hour after the reduction The image shows recurrent ileocolic intussusception to the level of the hepatic flexure (white arrows).

This patient was a three-year-old male with no previous past medical history who presented to the ED with 24 hours of intermittent colicky abdominal pain. without nausea or emesis. He presented with an upper respiratory infection earlier in the week. He remained hemodynamically stable, and on the examination, he had generalized abdominal tenderness with no guarding or rebound. Radiological workup included an unremarkable abdominal X-ray and an ultrasound showing ileocolic intussusception (Figure 6). The intussusception was successfully reduced with an air enema (Figure 7). However, one hour post-reduction, his abdominal pain returned. Repeat ultrasound revealed a recurrence of intussusception (Figure 8). At this time, this decision was made to proceed with surgical intervention. 

The patient underwent diagnostic laparoscopy, laparoscopic reduction of the intussusception. The bowel was healthy and viable. Since the appendix appeared dilated, the decision was made to proceed with an appendectomy. Pathology of the appendix showed lymphoid hyperplasia. Postoperatively, he did well and was discharged the next day.

## Discussion

Most cases of ileocolic intussusception are considered idiopathic, meaning that no specific abnormality is identified apart from hypertrophied lymphoid tissue [15]. Only a small percentage (approximately 5%) of cases can be attributed to a specific lead point, such as Meckel diverticulum, duplication cyst, tumor, or polyp. Within that 5%, the appendix as the lead point is a rare entity. In our first case, the lead point was identified to be an intussuscepted appendix, which also showed a tendency to invaginate back into the cecum.

Appendiceal intussusception was first reported by M’Kidd J in 1885 [16]. A 40-year study by Collins DC of 71,000 appendix specimens at autopsy revealed an incidence of appendiceal intussusception at just 0.01% [17]. The prevalence of appendiceal intussusception is found to be slightly higher in males than females in the pediatric population [18]. The appendix, whether in a normal or pathological state, can serve as a lead point for intussusception. While the appendix can intussuscept spontaneously, there are contributory attributes that make appendicular intussusceptions more likely, which can be broadly classified into anatomical or pathological factors [6].

Anatomical factors contributing to appendicular intussusception include a fully mobile appendix (when the appendix is highly mobile, it can move freely within the abdominal cavity, increasing the likelihood of it becoming a lead point for intussusception) and hyperperistalsis (excessive contractions of the intestinal muscles can also contribute to intussusception). Pathological conditions that can lead to appendicular intussusception include appendicular inflammation (inflammation of the appendix, such as in acute appendicitis, can cause swelling and changes in the shape and size of the organ, increasing the risk of intussusception), calcified fecalith (hardened mass of fecal matter that can obstruct the appendix and if calcified, it can act as a lead point for intussusception), benign conditions (various benign growths within the appendix, such as polyps, tubulovillous adenomas, mucoceles, and mucinous cystadenomas, can serve as lead points for intussusception), malignant conditions (certain types of cancers, such as mucinous cystadenocarcinoma, carcinoid tumors, and mucosa-associated lymphoma, can affect the appendix and lead to intussusception), and foreign bodies in the appendix (swallowed objects or ingested parasites can find their way into the appendix and trigger intussusception). 

It is important to note that while the appendix can be a potential lead point for intussusception, this condition is relatively uncommon compared to other causes of intussusception, such as intestinal tumors or inflammation in other parts of the gastrointestinal tract. Appendiceal intussusception also needs to be recognized when it presents as a filling defect within the cecum on enema reductions, which can be mistaken as a cecal obstructing mass [5].

Regarding the management of ICI with an appendix as the lead point, a review of the literature suggests that immediate surgical intervention and appendectomy are generally warranted. In our review, we found that enema reductions were associated with higher chances of recurrence when compared to surgical reduction with appendectomy [5,19-22]. Furlong et al. reported that even after five cycles of pneumatic enema reduction, the RICI persisted with the appendix inflamed and inverted within the cecum. Only after surgical reduction and appendectomy did the patient's symptoms finally resolve [5]. Similarly, Samuk et al. reported many unsuccessful attempts at manual reduction of recurrent ICI with the appendix as the lead point, which only resolved after a distal cecectomy and appendectomy [21]. Lipskar et al. suggested that when suggested by radiological imaging, immediate surgical reduction is preferred since enema reduction is rarely effective. However, surgical reduction and hydrostatic reductions can both lead to recurrence if done without an appendectomy [20]. This was also observed in our first case presentation, where treatment with pneumatic reduction quickly led to the recurrence of ICI with an appendix as the lead point. However, even with surgical reduction and appendectomy, the intussusception can still recur. Lipskar et al. described recurrence at the site of the appendectomy with the staple line as the lead point after laparoscopic appendectomy on POD four [20]. Subsequent laparoscopic partial cecectomy resolved all symptoms. A similar case also happened to our first case when the patient returned to the OR on POD four for invagination of the staple line with appendiceal stump at the site of the appendectomy into the cecum. Thus, in the cases of ICI where the appendix is involved, we recommend surgical intervention with manual reduction and appendectomy as a first-line treatment since non-operative reduction is rarely effective. In cases where the appendix is abnormal, a partial cecectomy near the base of the appendix should also be considered to reduce the risk of recurrence after appendectomy [23]. Overall, proper surgical intervention can potentially reduce medical costs and additional unnecessary ER visits, improving patient outcomes.

When discussing RICI, its treatment guidelines remain controversial: whether to undergo multiple enema reductions or if the surgical reduction is more beneficial. There is a paucity of literature describing criteria for surgical intervention of RICI, though some retrospective studies suggest nonoperative management with enema reduction should be the initial treatment for RICI [9]. Recurrence rate after enema reduction, up to 20%, is significantly higher compared to surgical reduction, 1% to 3% [24-26]. In episodes of recurrence, hydrostatic reduction and pneumatic reduction had a 96.2% and 92% success rate, respectively. In cases where surgery was done, the two-year follow-up was uneventful. In addition, pathologic lead points were found in 9.3% of patients who had RICI compared to 3.8% who did not have RICI [14]. Niramis et al. found that even when there is a pathologic lead point (PLP), it can be reduced by enema reductions [14], while Ein SH found that surgical management is necessary in the presence of a PLP [26]. 

It remains unclear after how many recurrences of intussusception is surgery indicated as this number differs from one to three [25,27,28]. Soper et al. recommend operative management after one episode of recurrence in patients older than two years [24] and after two episodes of recurrence for all ages [24,25]. Hsu et al. [13] and Chang et al. [29] suggest surgical reduction after three episodes of recurrence. Previously, it has been suggested that when surgical reduction is used, risks are lowered if used in primary intussusception [30,31]. Soper et al. claim that surgical reduction is necessary in order to determine the possibility of incomplete reduction, proximal invaginations, and specific etiologic lesions [24]. The most concerning complication of pneumatic reduction is intestinal perforation, but it has a low complication rate while surgery may not deliver a clear benefit [32]. Literature suggests that, overall, nonoperative management should be the first line for RICI, with surgical reduction being reserved for when there is a pathological lead point, such as the appendix in our cases, multiple recurrences, and in the presence of risks for enema reduction such as signs of perforation, shock, and peritonitis [3,9,27,28,32-38]. 

Our second case demonstrates an intervention that has historically not been first-line. Previously, studies have suggested that surgical reductions can be taken in cases where the patient is over the age of two years when a pathological lead point is suspected, delayed recurrence, and when there is more than one episode of recurrence [31]. In our case, swift recurrence of intussusception with thickening of bowel after pneumatic reduction prompted surgical intervention, which revealed a dilated appendix which served as the lead point for our patient’s recurrent intussusception. 

While Wang et al. showed that incidental, prophylactic, appendectomy performed during operative treatment for uncomplicated intussusception to eliminate future appendicitis as a diagnostic consideration is associated with increased length of stay (LOS) and adjusted total cost (ATC), further studies are needed to determine if routine appendectomy should be performed during surgical intervention for intussusception [38].

## Conclusions

Recurrent ileocolic intussusception is common but RICI with the appendix as the lead point is rare. These cases demonstrate the role of the appendix as a pathologic lead point and a review of literature supports the need for surgical reduction. While enema reduction is the first line for recurrent intussusception, surgical reduction is preferred when a pathological lead point is suspected such as the appendix.
